# Computational characterization of peptide binding stability to HLA-C allotypes and its association with HIV-1 infection progression and HIV-1 related neurocognitive impairment

**DOI:** 10.3389/fimmu.2025.1703026

**Published:** 2025-12-11

**Authors:** Mauro Voi, Antonella Sangalli, Erica Ginevra Milano, Carola De Martinis, Elisa Orlandi, Stefano Tamburin, Elisa Mantovani, Angela Federico, Massimiliano Lanzafame, Emanuela Lattuada, Gustavo Adolfo Argañaraz, Bosco Christiano Maciel Da Silva, Alberto Jose Da Silva Duarte, Jorge Casseb, Enrique Roberto Argañaraz, Marina Malena, Marco Albani, Alessandra Ruggiero, Maria Grazia Romanelli, Maria Teresa Valenti, Giovanni Grazioso, Donato Zipeto

**Affiliations:** 1 Department of Neurosciences, Biomedicine and Movement Sciences, University of Verona, Verona, Italy; 2 Department of Pharmaceutical Sciences, University of Milan, Milan, Italy; 3 Unit of Infectious Diseases, Santa Chiara Hospital, Azienda Provinciale per i Servizi Sanitari, Trento, Italy; 4 Centre for Medical Sciences (CISMED), University of Trento, Trento, Italy; 5 Laboratory of Molecular Neurovirology, Department of Pharmacy, Faculty of Health Science, University of Brasilia, Brasilia, Brazil; 6 Medical Investigation Laboratory Unit 56 (LIM/56), Faculdade de Medicina FMUSP, University of São Paulo, São Paulo, Brazil; 7 Faculty of Medicine, Institute of Tropical Medicine, University of São Paulo, São Paulo, Brazil; 8 U.O.S. Infectious Diseases, Santa Maria della Misericordia Hospital AULSS5, Rovigo, Italy

**Keywords:** HLA-C, HIV-1, antigen presentation, immune response, neurocognitive disorders, HAND, peptide-binding stability, personalized medicine

## Abstract

**Background:**

HLA-C molecules play a critical role in the immune response, particularly in antigen presentation and immune modulation.

**Methods:**

To investigate the effect of the most common HLA-C allotypes on the stability of the HLA-C-β-2 microglobulin-peptide complex, we used the NetMHCpan-4.2 bioinformatic tool that predicts peptide binding to MHC class I molecules. This allowed us to predict the probability of a broad set of peptides to be naturally processed, presented on each HLA-C allotype, and ultimately recognised by the immune system, measured by EL-score. By plotting the EL-score against the percentile of the peptide's stability rank position, curves were drawn to illustrate the relative stability of the binding interaction of each HLA-C allotype tested, and the area under the curve was calculated to determine a stability score for each HLA-C variant.

**Results:**

This approach permits us to greatly improve the classification of HLA-C allotypes according to their stability, overcoming the previous coarse stable and unstable binary classification. Analysis of two well-characterised HIV-1 patient cohorts, one focused on disease progression and the other on neurocognitive impairment, demonstrated a significant association between unstable HLA-C alleles, faster disease progression, and worse HIV-associated neurocognitive outcomes.

**Conclusions:**

These findings underscore the role of HLA-C stability in AIDS progression, suggesting that profiling HLA-C stability may serve as a predictive tool for HIV-1 disease management and assessing neurocognitive risk, with potential implications in personalised medicine.

## Introduction

The human leukocyte antigen (HLA) system is a key component of the innate and adaptive immune responses, playing a critical role in antigen presentation (1, 2). HLA-C is a class I major histocompatibility complex (MHC-I) molecule involved in the immune surveillance against infections and malignancies. Unlike HLA-A and HLA-B, HLA-C exhibits lower surface expression and distinct peptide-binding properties due to less efficient assembly and cell membrane expression (3–5), making its functional stability a critical aspect of immune response regulation (6). Despite its historically perceived lower relevance, emerging evidence has demonstrated that HLA-C plays a significant role in modulating immune responses, particularly in HIV-1 infection, influencing viral replication, immune escape mechanisms, and disease progression (7).

HLA-C is central to antigen presentation to CD8+ T cells and immune surveillance. Its genetic variability influences disease susceptibility and transplant compatibility (1, 2). Furthermore, the stability of the HLA-C-β-2 microglobulin (β_2_m)-peptide complex determines antigen presentation efficiency, with less stable variants linked to impaired immune responses, increased infection risk, and reduced immunotherapy efficacy (7–9).

HLA-C expression varies significantly among individuals (10) and is modulated by genetic factors, including promoter polymorphisms, microRNA interactions, and alternative splicing (9, 11–13). Single-nucleotide polymorphisms (SNPs) in the 3’ untranslated region (3’UTR) of the HLA-C gene affect its expression levels, particularly through regulation by miR-148a (14). Furthermore, HLA-C upregulation has been linked to greater immune pressure on HIV-1, driving viral evolution and immune escape mutations (10).

The efficiency of antigen presentation is directly linked to the stability of the HLA-C/peptide complex, which varies across alleles and can impact immune recognition and viral control. Unlike HLA-A and HLA-B, HLA-C displays more selective peptide-binding preferences, which impact its effectiveness in presenting viral antigens. HLA-C allotypes display considerable variation in their peptide-binding clefts, which directly influences peptide binding stability (9). These differences affect the capacity of HLA-C molecules to stabilize peptide-MHC complexes and maintain antigen presentation on the cell surface. While allotypes like HLA-C*05 and HLA-C*08 are characterized by higher binding stability, others, such as HLA-C*07, are more prone to peptide dissociation and degradation (6). Sibilio and colleagues evaluated the post-assembly stability of HLA-C variants using pulse-labelling assays in homozygous cell lines expressing eight serologically defined HLA-C alleles. Their findings revealed that some HLA-C variants, such as HLA-C*05, C*06, C*08, exhibit stronger binding to β_2_m, whereas others, including HLA-C*04 and C*07, display weaker β_2_m association and reduced complex stability (15).

The extensive polymorphism of HLA alleles presents challenges for experimentally characterizing HLA/peptide stability across variants *in vitro*. To overcome this limitation, several computational tools have been developed to predict the interaction between HLA and peptides (16), among which NetMHCpan stands out for its high predictive accuracy (17). This model incorporates multiple parameters, including binding affinity, stability, length, processing and presentation pathways, to estimate peptide presentation and immunogenicity (17). NetMHCpan uses artificial neural networks trained on experimental binding affinity and mass spectrometry-eluted ligand (EL) data to significantly improve the accuracy of peptide-binding predictions across different HLA alleles. Evolving through versions 4.0, 4.1 and most recently 4.2, it incorporates enhanced machine learning techniques and new training datasets to improve performance in identifying immunogenic peptides (17–19). A key innovation in recent versions is the integration of structural features to enhance the predictive power of the algorithm (18), as well as the EL-score (19), which incorporates data on naturally processed and presented peptides obtained via mass spectrometry-based ligand elution assays (20). This overcomes the limitations of the traditional IC50-based calculation of binding affinity. Therefore, the EL-score represents a composite measure that integrates binding affinity (BA), the traditional indicator of peptide-MHC interaction strength, with eluted ligand data from naturally presented peptides, enhancing biological relevance. It also incorporates peptide stability and processing parameters, reflecting the likelihood that a bound peptide remains stably displayed on the cell surface. The EL-score aims to predict the likelihood that a peptide will be naturally processed, presented on an MHC class I molecule, and ultimately trigger immunity. Studies have shown that the EL-score provides a better correlation with immunogenicity than BA alone. For instance, Harndahl et al. demonstrated that peptide-MHC stability is a stronger predictor of T cell activation than affinity, supporting the inclusion of EL data in prediction models (8). Similarly, Rasmussen et al. reported that combining affinity and stability scores improves the identification of CTL epitopes, reinforcing NetMHCpan’s value in epitope discovery for vaccine development (21). Recent improvements using deep learning frameworks and large-scale mass spectrometry have further refined EL-score predictions, supporting it as a robust metric linking computational models to experimental outcomes.

HLA-C expression levels and the stability of its association with β_2_m and peptide are key factors influencing antiviral immunity. Certain HLA-C alleles are linked to better control of HIV-1 replication (10). Variations in viral load associated with different HLA-C alleles are thought to result from differences in their capacity to present HIV-derived peptides to CD8+ T cells and NK cells—key players in targeting infected cells (22). Moreover, research has shown that HLA-C expression levels and peptide-binding stability are critical determinants of immune control (23).Unstable HLA-C variants have also been linked to increased HIV-1 infectivity. Previous findings from our group indicate that these variants may dissociate from β_2_m, forming free heavy chains that interact with the HIV-1 envelope glycoprotein (Env), enhancing viral infectivity (24). In contrast, stable HLA-C variants remain bound to β_2_m and retain strong antigen-presenting functions, which are associated with better immune control. Thus, binding stability to peptides confers to HLA-C the ability to act as a conventional molecule involved in cellular immunity, or as an accessory factor modulating HIV-1 infectivity (25).

HIV-associated neurocognitive disorders (HAND) encompass a spectrum of subjective and objective cognitive impairments, from mild to severe, that occur in approximately 50% of HIV-infected individuals and persist despite effective antiretroviral therapy, suggesting that host genetic factors influence their onset and progression (26). HLA-C polymorphism also plays a role in HAND (27, 28). Unstable HLA-C variants may contribute to higher levels of free β_2_m in cerebrospinal fluid, leading to chronic neuroinflammation and neuronal damage (29). This mechanism is supported by associations between high β_2_m levels and neurodegenerative conditions, including Alzheimer’s disease (30). Additionally, HLA-C*07 has been specifically linked to a higher incidence of HAND in HIV-positive individuals, further supporting the hypothesis that HLA-C variation plays a role in neurocognitive impairment (31).

In this study, we sought to thoroughly investigate HLA-C stability to evaluate its relevance to HIV-1 clinical outcomes. To these aims, we integrated bioinformatic predictions with clinical progression and neurocognitive outcomes.

## Materials and methods

### Molecular dynamics analysis

Starting from the crystal structures of five human allelic variants of HLA-C currently available on Protein Data Bank (specifically: HLA-C*03:04 - PDB ID: 1EFX; HLA-C*04:01 – PDB ID: 1QQD; HLA-C*05:01 – PDB ID: 5VGD; HLA-C*06:02 – PDB ID: 5W6A; HLA-C*07:02 – PDB ID: 5VGE), single point mutations were introduced on the co-crystallized nonapeptides using Maestro’s Workspace tool ‘Mutate Residue’ (release 2021–2, Schrödinger, LLC, New York, NY, USA). Subsequently, ff19SB force field (32) were applied on the systems using the “Protein Preparation Wizard” available in Maestro (release 2021–2, Schrödinger, LLC, New York, NY, USA), then the complexes were immersed in TIP3P (33) water cubic boxes and their geometry was energy minimized, allowing the remainder of the system (HLA-C and β_2_m) to adapt to the newly introduced oligopeptide sequences. Then, Molecular Dynamics (MD) simulations were accomplished on these systems, using the Amber24. The parameters for these MD simulations were configured as follows: 300 ns, 300 K, and 1 atm. For each system, we carried out three independent replicas. The attained trajectories were clustered, by means of Amber24’s cpptraj tool (34), to identify the different families of complex conformations and to identify the most populated ones. On these, the Molecular Mechanics-Generalized Born Surface Area (MM-GBSA) approach was applied to decompose the pairwise binding energetic contributions within the HLA/β_2_m interactions.

### Peptide and HLA-C stability binding analysis

**Figure 1:**
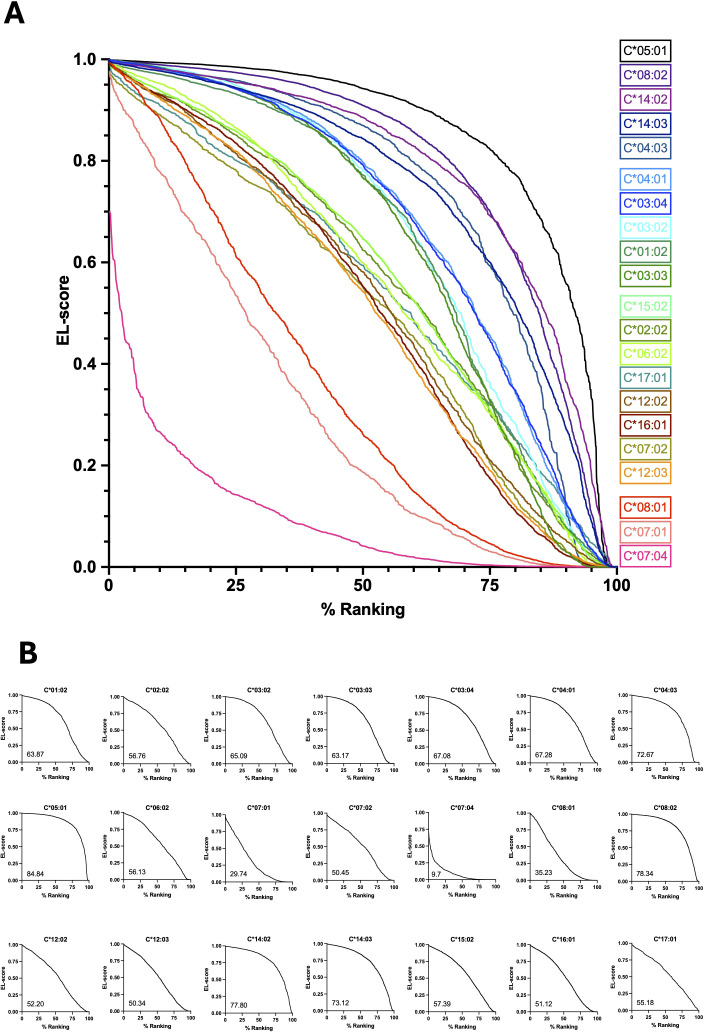
EL-score vs percentile ranking curves for each HLA-C/peptide pool. Individual EL-score values of each specific HLA-C allotype and its corresponding peptides, as predicted by NetMHCpan-4.2, were ranked and plotted against the percentile of the peptides’ ranking position, resulting in distinct stability distribution profiles for each HLA-C allele. HLA-C *05:01, C*08:02, C*14:02, C*14:03 and C*04:03 clearly show a strong binding trend with most peptides, whereas HLA-C*07:04, C*07:01 and C*08:01 exhibit the lowest EL-score values for most peptides. The curves are shown overlapped in **(A)**, where each curve is color-coded according to the corresponding HLA-C allotype, and separately in **(B)**, where the area under the curve for each HLA-C allotype is reported in the lower left corner of each plot.

Peptides binding to the 21 most frequent HLA-C allotypes in the human population (C*01:02, C*02:02, C*03:02, C*03:03, C*03:04, C*04:01, C*04:03, C*05:01, C*06:02, C*07:01, C*07:02, C*07:04, C*08:01, C*08:02, C*12:02, C*12:03, C*14:02, C*14:03, C*15:02, C*16:01, C*17:01) were experimentally validated by Sarkizova et al. (35). The final number of selected peptides was 36070, with a median of 1403 (range 730-3311) (**Table 1**). Their database cumulatively covers 95.8% of individuals worldwide based on allele frequencies. For our analysis, we focused on peptides of 8–12 amino acids, as they comprised most of the initial list and were the ones preferentially bound by MHC-I complexes (36). Allele frequencies were reported according to Sarkizova et al. (35). The Eluted Ligand (EL) score for each peptide binding to its specific HLA-C allotype was determined using NetMHCpan-4.2 (https://services.healthtech.dtu.dk/services/NetMHCpan-4.2/) with default parameters. Peptides were then ranked by NetMHCpan-4.2 EL-score for each HLA-C allele. The ranking was expressed in percentiles (% Ranking) to account for different peptide pool sizes. The resulting EL-score versus % Ranking curves were used to calculate the area under the curve (AUC) to determine a stability score for each HLA-C allotype considered (**Figure 1**).

**Table 1:** The most frequent human HLA-C allotypes and their calculated stability score.

Allotype	Peptides number	Frequency	Stability score (AUC)	Weighted average
C*01:02	1357	0.085	63.87	–
C*02:02	930	0.028	56.76	–
C*03:02	1194	0.025	65.09	*65.52*
C*03:03	2123	0.056	63.17	
C*03:04	2356	0.091	67.08	
C*04:01	1854	0.112	67.28	*68.06*
C*04:03	1038	0.019	72.67	
C*05:01	1442	0.026	84.84	*-*
C*06:02	1324	0.062	56.13	*-*
C*07:01	808	0.069	29.74	*40.96*
C*07:02	1072	0.131	50.45	
C*07:04	730	0.015	9.70	
C*08:01	1802	0.045	35.23	*-*
C*08:02	3148	0.020	78.34	*-*
C*12:02	1403	0.032	52.20	*-*
C*12:03	2175	0.020	50.34	*-*
C*14:02	1371	0.025	77.80	*76.05*
C*14:03	2784	0.015	73.12	
C*15:02	3311	0.034	57.39	–
C*16:01	2883	0.024	51.12	–
C*17:01	965	0.019	55.18	–

For the 21 most frequent HLA-C allotypes (first column), the number of specific binding peptides (8-12mer) identified by Sarkizova et al. (35) is reported (second column) alongside their frequency in the human population (third column). The area under the curve (AUC) calculation (fourth column) provides a stability score for each allotype. When second-digit typing was not possible, an average stability score weighted by frequencies was calculated (fifth column).

### Patient cohorts

Two groups of HIV-1 infected individuals were enrolled in this study and categorized based on disease progression and neurocognitive status.

The first cohort included 47 patients classified as progressors (P; >10,000 copies HIV genome/mm^3^, and ≤200 CD4+ T lymphocytes/mm^3^) and 37 patients classified as long-term non-progressors (LTNPs; <10,000 copies HIV genome/mm^3^, and ≥400 CD4+ T lymphocytes/mm^3^). HIV viral-load measurements were obtained periodically throughout each patient’s follow-up period (defined as the interval between initial infection detection and the end of monitoring). LTNPs were followed for a minimum of seven years. Progressors were observed until their CD4^+^ T-cell count declined to ≤200 cells/mm³, at which point antiretroviral treatment was initiated and sample collection was discontinued. All subjects gave informed consent, and the research protocols were approved by the relevant institutional review boards and research ethics committees (Ethics Committee of the Health Department of the Federal District (#066/07); and the Ethics Committee for Analysis of the public network of the Federal District, Brazil; and the Ethics Committee for Analysis of Research Projects (CAPPesq) of Hospital das Clínicas HCFMUSP (#CAPPesq #0306/10, Online registration #5867), from the Faculty of Medicine at the University of São Paulo, Brazil. The cohort is unique since the study participants had not received any treatment at the time of sampling. Further details on the patient cohort are reported in our previous study (37).

The second cohort included 57 patients referred by the Infectious Diseases Outpatient Clinics of the Verona University Hospital a) aged < 67 years, b) on dual/triple antiretroviral therapy regimens, and with c) stable suppressed plasma viremia. Participants with a history of recurrent drug abuse, current drug addiction, or other neurological diseases (e.g., history of cerebrovascular events) that could cause cognitive decline were excluded. Each patient underwent a comprehensive neuropsychological test battery designed to measure the cognitive domains recommended by the three main diagnostic guidelines (i.e., attention, executive function, learning and memory, language, speed of processing, complex motor skills) (26). The impact of cognitive difficulties on the ability to perform everyday activities was also assessed. As neuropsychiatric symptoms frequently occur alongside HIV infection, anxiety and depression were evaluated. The Frascati criteria (38) were used as the gold standard for diagnosing HIV-associated neurocognitive disorders (HAND) and for dividing patients into HAND-positive (n=16) and HAND-negative (n=41) groups. Patients reporting subjective cognitive complaints were added to the first group, in line with the most recent recommendations provided by the International HIV-Cognition Working Group, which highlight the importance of changes in cognition that have been noticed by the individual or an observer, even in the absence of impact on daily functioning (39). All patients signed an informed consent for the study that was conducted according to the Declaration of Helsinki and approved by the Institutional Ethics Committee of Verona and Rovigo (Italy) (#2459 CESC).

### HLA-C genotyping and sequencing

DNA samples from all patients were extracted from peripheral blood lymphocytes and subjected to allele-specific polymerase chain reaction (AS-PCR) and Sanger sequencing to determine HLA-C allotypes as described in our previous study (37). HLA-C allotypes C*01, C*02, C*05, C*06, C*12, C*14, C*15, C*16 and C*17 were typed at one digit resolution, since less common allotypes were mostly clustering in the same subgroup according to Shen et al. (40). Allele-specific PCR was used to type C*12:03 and C*16:01. Sequencing analysis of the HLA-C region between exons 2 and 3 was utilized to further characterize HLA-C*03:02, C*03:03, C*03:04, C*04:01, C*04:03, C*07:01, C*07:02, C*07:04, C*08:01 and C*08:02 variants at second digit resolution, by performing sequence alignments at the Immuno Polymorphism Database (IPD https://www.ebi.ac.uk/ipd/index.html).

### Determination of patient-specific HLA-C stability score

A stability score was determined for each patient based on their HLA-C genotype by multiplying the allotype stability scores (determined by the AUC of the corresponding allotypes). For HLA-C alleles typed at the first-digit resolution, the stability score of the most frequent allotype was used.

For HLA-C*14, the two most common allotypes (C*14:02 and C*14:03) have similar frequencies (35) and are clustered within the same subgroup, according to Shen et al. (40). In this case, the allotype stability score was determined by calculating the frequency-weighted mean. Similarly, when a patient’s DNA was insufficient for second-digit genotyping, the frequency-weighted mean was calculated.

Finally, since allele-specific PCR for HLA-C*12 only identifies the common subtype C*12:03 at the second-digit, the stability score of the other most frequent C*12 allotype (C*12:02) was assigned when C*12 typing did not match C*12:03.

### Statistical analysis

Statistical analyses were performed using GraphPad Prism (version 10). Comparisons between independent patient groups (LTNP vs P; HAND- vs HAND+) were performed using the two-tailed Mann–Whitney U test. A p-value < 0.05 was considered statistically significant.

## Results

### HLA-C allotypes exhibit different binding stability with their specific peptides

The analysis of the EL-score distribution revealed significant variations in HLA-C binding stability. Indeed, allele C*05:01 showed a strong binding for most peptides, while peptides specifically binding allele C*07:04 presented much lower binding prediction values. The determination of a stability score by calculating the AUC allowed a clear identification of different stability values describing the binding of each allele to its own peptide pool. Accordingly, some alleles, such as HLA-C*07:04, C*07:01 and C*08:01, displayed weak binding interactions, while others, such as HLA-C*05:01, C*08:02 and C*14, exhibited strong binding stability (**Figure 1**). Notably, we identified discrepancies within allele subtypes that challenged previous classifications of stability. For example, HLA-C*07, previously considered an unstable allele (37), showed subtype-dependent variability, with C*07:02 demonstrating a greater stability score than C*07:01 and C*07:04. Similarly, HLA-C*08, considered a stable allele, presented a similar subtype variability, with C*08:02 among the variants with the highest stability, but C*08:01 among those with the lowest. The considered HLA-C allotypes and the calculated stability scores are reported in **Table 1**.

### The HLA-C complex stability is determined by the interactions with the peptide

**Figure 2:**
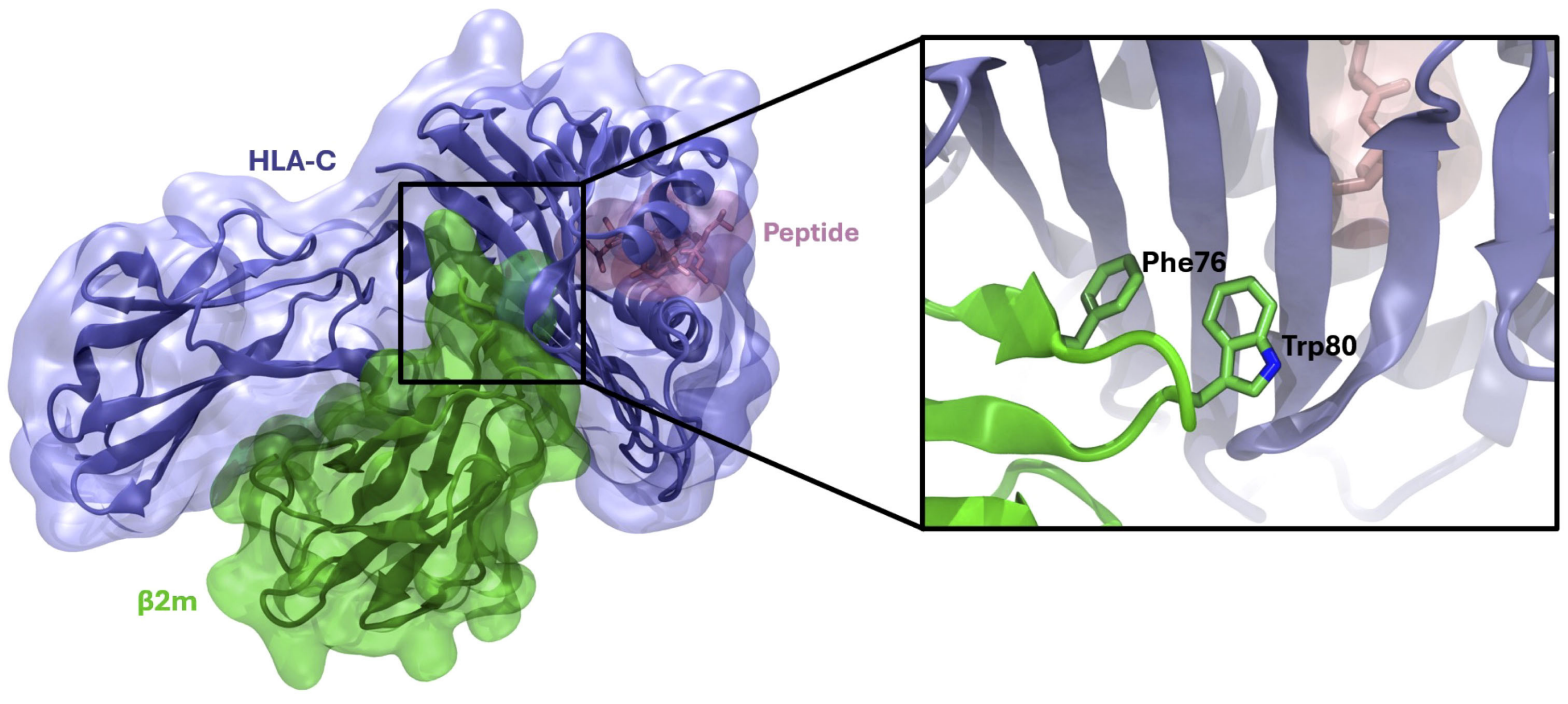
HLA-C/β_2_m/peptide complex. A 3D representation of the HLA-C/β_2_m/peptide complex, as obtained at the end of MD simulations, is shown on the left. On the right, a detailed visualization of the HLA-C/β_2_m interface, highlighting residues critical for mediating protein-protein interactions, is shown.

Computational studies were performed to acquire atomistic details on the interaction between HLA-C/peptide and β_2_m. Analyses were performed after selecting a group of HLA-C variants for which the crystal structures were available in the Protein Data Bank (www.rcsb.org). We randomly selected two 9-mer peptides specific for each allotype tested. After accomplishing MD simulations of the selected peptides in complex with the HLA-C/β_2_m heterodimer, the analysis of the MD trajectories and the data retrieved from the pairwise energy decomposition revealed that there was a clear recurring pattern in β_2_m’s “hot spot” residues involved in the interaction with HLA-C. In fact, the trend of the residues mainly contributing and their energetic contributions’ values were superimposable within the different allelic variants, but also amongst the different peptides (**Table 2**). On average (**Figure 2** and **Table 2**), our analysis revealed that a) β_2_m-Trp80 and -Trp61 give the main energetic contribution in each considered complex, since their energetic contribution’s values are always among -9 and -12 kcal/mol; b) β_2_m-Phe76 always stands in second position, with values around -5/-6 kcal/mol; c) The β_2_m-Tyr30 residue is another key contributor to the interaction, providing a consistent energetic contribution of around -4 to -5 kcal/mol across all complexes.

**Table 2:** Energetic contributions of β_2_m residues to HLA-C complex stability.

Allotype	Peptide	EL-score	% Ranking	β2m residue	ΔG [kcal/mol]
C*03:04	QATMPHLSM	0.610	64.941	Trp80	-10.1
				Phe76	-5.8
	TITDIISAL	0.712	57.598	Trp80	-10.5
				Phe76	-5.7
C*04:01	YHDKNIVLL	0.813	48.220	Trp80	-10.7
				Phe76	-4.7
	TFESLVAKL	0.533	70.712	Trp61	-9.4
				Phe76	-5.2
C*05:01	NLDQPPAFF	0.924	56.380	Trp80	-9.3
				Phe76	-5.6
	NAEAKITKL	0.621	88.141	Trp 61	-10.3
				Phe76	-5.5
C*06:02	FKMTIPLLV	0.490	59.063	Trp80	-11.7
				Phe76	-6.1
	VYYLKNREV	0.563	53.852	Trp80	-9.8
				Phe76	-6.5
C*07:02	LRHPVCVEL	0.439	61.007	Trp80	-10.4
				Phe76	-5.8
	FYRVTTEQY	0.499	55.877	Trp80	-10.7
				Phe76	-5.6

Binding free energy values (ΔG) of β_2_m residues that are functionally important for interaction were analyzed for five different HLA-C allotypes for which crystallographic structures are available in the Protein data bank (www.rcsb.org). The analysis was performed on the trimeric complex consisting of the HLA-C heavy chain, β_2_m, and peptide. Two randomly chosen peptides specific to each allotype were analyzed. The β_2_m residues primarily involved in interacting with HLA-C were Trp80 and Phe76. These residues formed comparable contacts and exhibited similar ΔG values across different HLA-C allotypes.

These data clearly indicate that β_2_m maintains an invariant binding pattern with HLA-C heavy chain molecules, with consistent binding affinity and interacting residues, regardless of the bound peptide or HLA-C subtype.

### Lower HLA-C stability scores are associated with HIV-1 progression and HIV-1-related neurocognitive impairment

Peptide specificity is determined by interactions of peptide side chains with six binding pockets in the HLA-C peptide-binding site (41). Kangueane et al. (42) assigned common HLA-C alleles to 18 different sub-supertypes, with variants within the same sub-supertype generally binding a highly shared set of peptides. It is thus possible to predict peptide binding of other members of a supertype using experimental results based on just one member of the type (42). A more recent classification by Shen et al. (40) proposed three main subtypes for HLA-C (C7 includes various C*07 alleles; C1 includes C*05, C*17 and most of C*01 and C*15; C2 includes C*02, C*06, C*14, C*16). Because structural clustering of the peptide-binding region correlates with binding specificity (40), according to the supertype classification by Shen et al., we adopted typing at the second-digit level for structurally divergent alleles belonging to different subtypes (for instance C*03:02 to C2, while C*03:03 and C*03:04 to C1) or to different subgroups within C1 (C*04:03 and C*04:01, or C*08:01 and C*08:02), C7 (C*07:01, C*07:02 and C*07:04) and C2 (C*12:02 and C*12:03) subtypes, in order to obtain a more precise identification of the specific allotype. Based on these considerations, HLA-C typing made it possible to calculate a stability score related to each patient’s genotype and to correlate this value to different outcomes of HIV-1 infection. The stability scores calculated based on the HLA-C genotype of each HIV-1 patient considered are shown in **Table 3** and **Table 4**.

**Table 3:** HLA-C stability scores in HIV-1 patients according to AIDS progression.

Sample code	HIV-1 progression	Sex	Age	HLA-C genotype and stability scores				Stability score
				1^st^ allele	Score (1^st^)	2^nd^ allele	Score (2^nd^)	
PR1	LTNP	M	42	*03	*65.52*	*08:02	78.34	5132.84
PR2	LTNP	M	29	*03	*65.52*	*03	*65.52*	4292.87
PR3	LTNP	F	36	*06	56.13	*07:02	50.45	2831.76
PR4	LTNP	M	54	*02	56.76	*12:03	50.34	2857.30
PR5	LTNP	F	47	*07:02	50.45	*12	52.20	2633.49
PR6	LTNP	M	23	*05	84.84	*05	84.84	7197.83
PR7	LTNP	M	55	*05	84.84	*06	56.13	4762.07
PR8	LTNP	M	35	*02	56.76	*06	56.13	3185.94
PR9	LTNP	M	58	*05	84.84	*08:02	78.34	6646.37
PR10	LTNP	M	46	*07:02	50.45	*14	*76.05*	3836.72
PR11	LTNP	M	32	*07:02	50.45	*12:03	50.34	2539.65
PR12	LTNP	M	26	*05	84.84	*12:03	50.34	4270.85
PR13	LTNP	M	33	*02	56.76	*15	57.39	3257.46
PR14	LTNP	M	52	*07:01	29.74	*08:02	78.34	2329.83
PR15	LTNP	M	42	*04:01	67.28	*07:01	29.74	2000.91
PR16	LTNP	M	34	*04:01	67.28	*07:02	50.45	3394.28
PR17	LTNP	M	44	*04:01	67.28	*08:02	78.34	5270.72
PR18	LTNP	M	55	*01	63.87	*12	52.20	3334.01
PR19	LTNP	M	32	*14	*76.05*	*08:02	78.34	5957.76
PR20	LTNP	M	49	*04:01	67.28	*08:02	78.34	5270.72
PR21	LTNP	F	42	*04:01	67.28	*16:01	51.12	3439.35
PR22	LTNP	F	54	*07:01	29.74	*12	52.20	1552.43
PR23	LTNP	M	51	*04:01	67.28	*08:02	78.34	5270.72
PR24	LTNP	F	58	*01	63.87	*07:02	50.45	3222.24
PR25	LTNP	F	77	*01	63.87	*04:01	67.28	4297.17
PR26	LTNP	M	39	*04:01	67.28	*06	56.13	3776.43
PR27	LTNP	M	40	*04:01	67.28	*12	52.20	3512.02
PR28	LTNP	F	43	*05	84.84	*02	56.76	4815.52
PR29	LTNP	F	47	*04:01	67.28	*06	56.13	3776.43
PR30	LTNP	M	28	*07:01	29.74	*05	84.84	2523.14
PR31	LTNP	F	49	*03	*65.52*	*02	56.76	3718.92
PR32	LTNP	M	53	*07	*40.96*	*16:01	51.12	2093.88
PR33	LTNP	M	30	*03:02	65.09	*06	56.13	3653.50
PR34	LTNP	M	49	*05	84.84	*06	56.13	4762.07
PR35	LTNP	F	43	*01	63.87	*06	56.13	3585.02
PR36	LTNP	M	27	*05	84.84	*12:03	50.34	4270.85
PR37	LTNP	M	57	*06	56.13	*12:03	50.34	2825.58
PR38	P	M	45	*04:01	67.28	*07:02	50.45	3394.28
PR39	P	M	57	*03	*65.52*	*07:01	29.74	1948.56
PR40	P	M	28	*06	56.13	*07:01	29.74	1669.31
PR41	P	M	41	*07:02	50.45	*12	52.20	2633.49
PR42	P	M	40	*02	56.76	*04:01	67.28	3818.81
PR43	P	M	42	*04:01	67.28	*07:01	29.74	2000.91
PR44	P	M	25	*04:01	67.28	*08:02	78.34	5270.72
PR45	P	M	23	*05	84.84	*07:01	29.74	2523.14
PR46	P	M	30	*03:02	65.09	*04	*68.06*	4430.03
PR47	P	M	30	*05	84.84	*07:01	29.74	2523.14
PR48	P	M	28	*07:02	50.45	*12:03	50.34	2539.65
PR49	P	M	31	*03	*65.52*	*07:01	29.74	1948.56
PR50	P	M	39	*02	56.76	*04:01	67.28	3818.81
PR51	P	M	37	*04:01	67.28	*07:02	50.45	3394.28
PR52	P	M	26	*03	*65.52*	*02	56.76	3718.92
PR53	P	M	36	*04:01	67.28	*04:01	67.28	4526.60
PR54	P	M	51	*07:04	9.7	*15	57.39	556.68
PR55	P	F	47	*04	*68.06*	*16	51.12	3479.23
PR56	P	M	28	*04:01	67.28	*06	56.13	3776.43
PR57	P	M	65	*07:04	9.7	*15	57.39	556.68
PR58	P	M	61	*07	*40.96*	*07	*40.96*	1677.72
PR59	P	F	30	*07:01	29.74	*04:01	67.28	2000.91
PR60	P	M	57	*04:01	67.28	*12:03	50.34	3386.88
PR61	P	M	41	*03:02	65.09	*07:02	50.45	3283.79
PR62	P	M	54	*07:02	50.45	*07:02	50.45	2545.20
PR63	P	M	24	*04	*68.06*	*05	84.84	5774.21
PR64	P	M	31	*03:02	65.09	*06	56.13	3653.50
PR65	P	M	30	*07:02	50.45	*08:02	78.34	3952.25
PR66	P	M	64	*01	63.87	*02	56.76	3625.26
PR67	P	M	48	*04:01	67.28	*15	57.39	3861.20
PR68	P	F	55	*07	*40.96*	*05	84.84	3475.05
PR69	P	F	41	*06	56.13	*16	51.12	2869.37
PR70	P	F	50	*07:02	50.45	*16:01	51.12	2579.00
PR71	P	M	30	*04:01	67.28	*08:02	78.34	5270.72
PR72	P	M	49	*03	*65.52*	*06	56.13	3677.64
PR73	P	F	32	*04:01	67.28	*06	56.13	3776.43
PR74	P	M	61	*03	*65.52*	*07:01	29.74	1948.56
PR75	P	M	29	*03:02	65.09	*07:02	50.45	3283.79
PR76	P	F	53	*04:01	67.28	*06	56.13	3776.43
PR77	P	M	57	*04	*68.06*	*12	52.20	3552.73
PR78	P	F	57	*04	*68.06*	*07:04	9.70	660.18
PR79	P	M	33	*07:01	29.74	*07:02	50.45	1500.38
PR80	P	M	32	*03	*65.52*	*15	57.39	3760.19
PR81	P	F	39	*04	*68.06*	*12:03	50.34	3426.14
PR82	P	F	32	*07	*40.96*	*16:01	51.12	2093.88
PR83	P	M	63	*06	56.13	*07:02	50.45	2831.76
PR84	P	F	50	*07	*40.96*	*17	55.18	2260.17

HIV-1 infected individuals who showed either slow (Long Term Non Progressors, LTNP) or rapid progression (P) to AIDS were genotyped for HLA-C alleles. The stability values corresponding to each allotype were multiplied together to give a final HLA-C stability score for each patient. Allele scores calculated using a frequency-weighted average are reported in italics. Patient code, sex (M, male; F, female), HLA-C alleles and their corresponding stability scores, and the final calculated stability scores are reported.

**Table 4:** HLA-C stability scores in HIV-1–infected patients with and without neurocognitive impairment.

Sample code	Neurocognitive status	Sex	Age	HLA-C genotype and stability scores				Stability score
				1^st^ allele	Score (1^st^)	2^nd^ allele	Score (2^nd^)	
H1	HAND +	M	42	*01	63.87	*06	56.13	3585.02
H2	HAND +	F	46	*03:02	65.09	*07:02	50.45	3283.79
H3	HAND +	M	60	*02	56.76	*16:01	51.12	2901.57
H4	HAND +	M	59	*03:02	65.09	*04:01	67.28	4379.26
H5	HAND +	F	52	*07:02	50.45	*07:04	9.70	489.37
H6	HAND +	M	66	*03:02	65.09	*16:01	51.12	3327.40
H7	HAND +	M	40	*06	56.13	*07:01	29.74	1669.31
H8	HAND +	M	63	*01	63.87	*07:01	29.74	1899.49
H9	HAND +	M	56	*04:01	67.28	*07:01	29.74	2000.91
H10	HAND +	F	53	*06	56.13	*12:03	50.34	2825.58
H11	HAND +	M	52	*05	84.84	*07:01	29.74	2523.14
H12	HAND +	M	56	*04:01	67.28	*12:03	50.34	3386.88
H13	HAND +	M	51	*07:01	29.74	*07:01	29.74	884.47
H14	HAND +	F	65	*02	56.76	*07:01	29.74	1688.04
H15	HAND +	M	58	*07:01	29.74	*12:03	50.34	1497.11
H16	HAND +	M	64	*07:01	29.74	*08:02	78.34	2329.83
H17	HAND -	M	57	*07:01	29.74	*07:02	50.45	1500.38
H18	HAND -	M	58	*07:01	29.74	*15	57.39	1706.78
H19	HAND -	F	54	*07:01	29.74	*14	*76.05*	2261.73
H20	HAND -	M	45	*04:01	67.28	*15	57.39	3861.20
H21	HAND -	M	31	*07:01	29.74	*12:03	50.34	1497.11
H22	HAND -	F	53	*02	56.76	*12:03	50.34	2857.30
H23	HAND -	M	39	*03:02	65.09	*12:03	50.34	3276.63
H24	HAND -	F	56	*04:01	67.28	*06	56.13	3776.43
H25	HAND -	M	41	*04:01	67.28	*12:03	50.34	3386.88
H26	HAND -	M	55	*04:01	67.28	*04:01	67.28	4526.60
H27	HAND -	F	56	*05	84.84	*12:03	50.34	4270.85
H28	HAND -	NA	NA	*04:01	67.28	*07:01	29.74	2000.91
H29	HAND -	M	62	*05	84.84	*07:01	29.74	2523.14
H30	HAND -	F	57	*07:02	50.45	*12:03	50.34	2539.65
H31	HAND -	M	47	*07:02	50.45	*15	57.39	2895.33
H32	HAND -	M	60	*02	56.76	*15	57.39	3257.46
H33	HAND -	M	60	*06	56.13	*07:01	29.74	1669.31
H34	HAND -	M	62	*03:02	65.09	*07:02	50.45	3283.79
H35	HAND -	M	59	*08:02	78.34	*17	55.18	4322.80
H36	HAND -	M	65	*01	63.87	*14	*76.05*	4857.31
H37	HAND -	M	62	*06	56.13	*12:03	50.34	2825.58
H38	HAND -	M	37	*01	63.87	*07:01	29.74	1899.49
H39	HAND -	M	54	*02	56.76	*08:02	78.34	4446.58
H40	HAND -	F	64	*04:01	67.28	*07:02	50.45	3394.28
H41	HAND -	M	59	*04:01	67.28	*06	56.13	3776.43
H42	HAND -	M	46	*07:02	50.45	*14	*76.05*	3836.72
H43	HAND -	M	64	*04:01	67.28	*06	56.13	3776.43
H44	HAND -	F	54	*05	84.84	*16:01	51.12	4337.02
H45	HAND -	M	58	*05	84.84	*07:01	29.74	2523.14
H46	HAND -	M	54	*07:01	29.74	*07:02	50.45	1500.38
H47	HAND -	M	55	*03:02	65.09	*14	*76.05*	4950.09
H48	HAND -	M	54	*06	56.13	*06	56.13	3150.58
H49	HAND -	M	47	*04:01	67.28	*07:02	50.45	3394.28
H50	HAND -	M	60	*04:01	67.28	*06	56.13	3776.43
H51	HAND -	M	51	*03:02	65.09	*12:03	50.34	3276.63
H52	HAND -	M	64	*04:01	67.28	*04:01	67.28	4526.60
H53	HAND -	M	65	*03:02	65.09	*12:03	50.34	3276.63
H54	HAND -	M	47	*04:01	67.28	*05	84.84	5708.04
H55	HAND -	M	48	*04:01	67.28	*16:01	51.12	3439.35
H56	HAND -	M	45	*07:01	29.74	*15	57.39	1706.78
H57	HAND -	M	53	*04:01	67.28	*07:01	29.74	2000.91

HIV-infected individuals presenting (HAND-positive, HAND+) on not presenting (HAND-negative, HAND-) neurocognitive impairment were genotyped for HLA-C alleles. For each patient, the stability score of each allele was multiplied to obtain the final HLA-C stability score. Allele scores calculated using a weighted average are reported in italics. Patient code, sex (M, male; F, female; NA, not available), HLA-C alleles and their respective stability scores, and the final calculated stability scores are reported.

**Figure 3:**
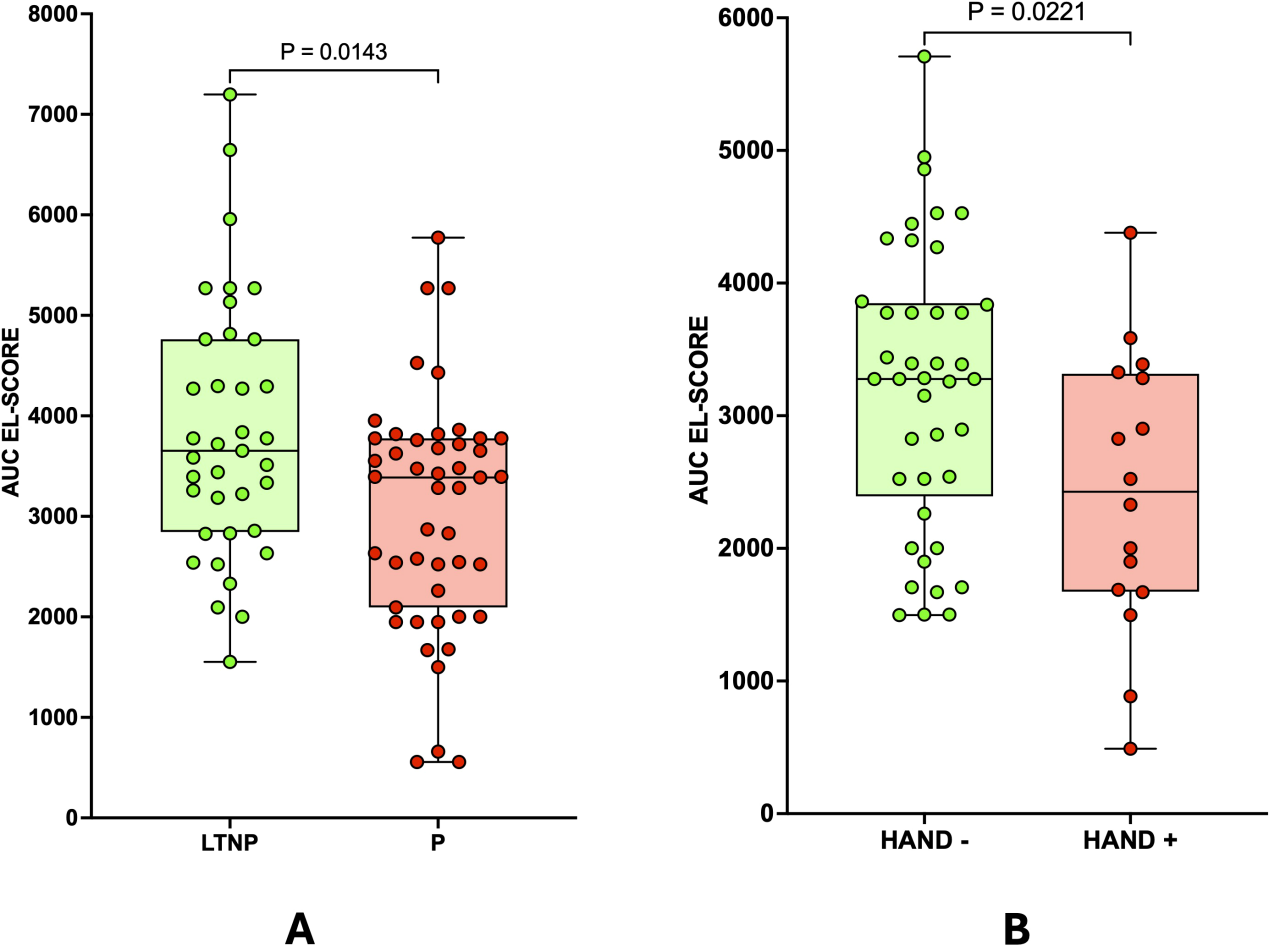
Association of patient HLA-C stability scores with HIV-1 progression and HIV-1-related neurocognitive impairment. **(A)** HIV-1 patients who experience rapid disease progression (Progressors, P, n=47) exhibit lower stability scores than those with slower progression (Long Term Non Progressors, LTNP, n=37). **(B)** HIV-1 patients presenting neurocognitive impairment (HAND+, n=16) have lower stability scores than cognitively normal patients (HAND-, n=41). Statistical analyses were performed using the two-tailed Mann-Whitney U test; p < 0.05 was considered statistically significant.

We found that Progressors (P) exhibited significantly lower stability scores compared to Long Term Non Progressors (LTNP) (p = 0.0143), supporting the hypothesis that unstable HLA-C alleles are associated with more severe disease outcomes (**Figure 3A**).

The examination of neurocognitive outcomes showed that HAND-positive patients had a higher prevalence of unstable alleles, with a statistically significant difference in stability scores compared to HAND-negative patients (p = 0.0221) (**Figure 3B**).

## Discussion

We explored HLA-C stability by means of advanced computational techniques and found that HLA-C stability influenced HIV-1 progression and cognitive outcomes. The identification of allele subtypes with different stability levels challenges traditional classifications of HLA-C as simply “stable” or “unstable,” highlighting the advantages of a more precise and individualized approach in assessing HLA-C function. Moreover, our findings provide compelling evidence that HLA-C stability may contribute to shaping both immune responses and neurological outcomes in HIV-1 infected individuals.

### HLA-C function is related to its stability

HLA-C stability is significantly influenced by genetic variations, which can either enhance or reduce its functionality within the immune response system (43, 44). HLA-C molecules exhibit differential stability based on genetic variations in their promoter regions, affecting their expression levels and antigen presentation efficiency. Some alleles, e.g., HLA-C*05, exhibit high stability and remain on the cell surface for prolonged surface expression, whereas others, e.g., HLA-C*07, display lower stability, impairing peptide loading and presentation (9). These observations are consistent with previous findings showing that inefficient peptide binding contributes to lower surface expression of HLA-C compared to HLA-A and HLA-B (6), likely due to its greater selectivity for peptide binding (3).

Several experimental and computational methodologies have been employed to assess HLA-C peptide binding stability. Mass spectrometry-based ligand elution assays have been used to profile naturally processed peptides, revealing a preference for 9-mer peptides in HLA-C binding (35, 36). Computational tools such as NetMHCpan and MHCflurry have demonstrated increased predictive accuracy in assessing binding affinities and stability of peptide-HLA interactions (19). Notably, peptide-MHC stability, rather than binding affinity alone, has been identified as a better predictor of T-cell immunogenicity (8). Additionally, computational tools like NetMHCpan and mass spectrometry-based ligand profiling have significantly improved the prediction and validation of HLA-C peptide-binding stability (19). These methodologies may provide insights into how stability affects immune function, disease progression, and therapeutic outcomes.

Recent studies have shown that HLA-C expression varies widely in an allele-specific manner, with higher expression levels exerting greater selection pressure on HIV-1, leading to virus-mediated downregulation of HLA-C (9). Certain single-nucleotide polymorphisms (SNPs) in the promoter and 3’ untranslated region (UTR) of HLA-C, such as rs9264942 (located in the 5’ UTR of the HLA-C gene), have been correlated with HIV viral load and disease progression (45). Additionally, the 3’UTR of HLA-C is a target for microRNA regulation (miR-148a), which influences HLA-C surface expression (14). This regulatory mechanism plays a crucial role in determining antigen presentation efficiency and immune escape strategies employed by HIV-1.

Our computational analysis supports evidence that HLA-C allotypes differ in their ability to bind and stabilize peptides, influencing their expression levels on the cell surface. Structural studies have demonstrated that HLA-C*07 has a deeper and narrower antigen-binding cleft, while HLA-C*05 has a relatively flat peptide-binding groove, allowing it to bind a broader range of peptides and remain more stably expressed on the cell membrane (9). These findings align with our stability analysis, which demonstrated that HLA-C*07:04 is the most unstable allele, while HLA-C*05:01 exhibits the highest stability. Importantly, the inefficient association of certain HLA-C variants with β_2_m leads to the accumulation of misfolded HLA-C heavy chains, further affecting antigen presentation efficiency (6, 15).

In addition, we noted that some very unstable variants, such as C*07:01 or C*07:04, are also the lowest expressed variants, whereas some more stable variants, such as C*05:01 or C*14:02, are among the most highly expressed alleles. While numerous factors contribute to the regulation of HLA-C expression levels, the peptide-binding capacity of different HLA-C variants is also recognized as a key determinant influencing their surface expression (46). Therefore, it is plausible that inefficient peptide binding may contribute to the lower expression of certain HLA-C variants on the cell membrane (9), suggesting a potential correlation between stability and expression levels.

Our analysis improved the definition of the stability of HLA-C allotypes, overcoming the previous binary classification, which was too simplistic and inaccurate. The in-depth characterization of binding stability to peptides specific to the most common variants in the human population was carried out using pools of experimentally validated peptides known to bind to the main allotypes (35). This approach enabled obtaining a “stability coefficient” for each of these HLA-C variants and thereby quantified the overall stability profile of each HLA-typed individual based on the combination of their specific allotype stability coefficients.

### HLA-C stability influences HIV-1 infection progression

HLA-C stability plays a critical role in modulating defense mechanisms against HIV-1. Unstable alleles may increase HIV-1 progression, suggesting that by influencing antigen presentation efficiency, they may alter the ability of CD8+ T cells to recognize and eliminate infected cells, in keeping with previous findings indicating that HLA-C expression levels directly affect HIV-1 immune control (10). Additionally, in our previous studies (24, 25) we reported that stable HLA-C alleles are associated with lower viral loads and more effective immune responses, supporting our finding that unstable alleles contribute to a faster progression to AIDS.

To assess the correlation between stability metrics and progression of HIV-1 infection, we re-analyzed a population of HIV-1 positive, treatment-naïve subjects described and characterized in our previous study (37). In the original analysis, the different allotypes were binary divided into stable and unstable, revealing a significant correlation between rapid progression to AIDS and the presence of unstable HLA-C variants. In the present study, we refined the analysis by performing high-resolution typing of the second digit for divergent subtypes and assessing a quantitative stability coefficient based on their HLA-C allotype combination. This enhanced approach confirmed and strengthened our earlier findings, showing a robust and highly significant correlation between accelerated disease progression and a higher burden of unstable HLA-C allotypes.

### HLA-C stability impact on HIV-associated neurocognitive outcomes

The association between unstable HLA-C alleles and HAND is particularly noteworthy. Previous studies have suggested that unstable HLA-C variants may lead to increased levels of free β_2_m, contributing to neuroinflammation and neuronal damage (29). Additionally, a recent study has specifically linked HLA-C*07 to HAND in HIV patients (31).

To test the clinical relevance of the specific stability coefficient for each major HLA-C allotype on neurological outcomes in HIV-1 infection, we analyzed a population of HIV-positive subjects with subjective report or objective evidence of cognitive impairment and compared them to cognitively unimpaired ones. We observed a significantly higher presence of unstable HLA-C variants in HAND-positive subjects than in HAND-negative ones, further confirming our preliminary observation on a small case series (29). Our findings confirm this association, suggesting that HLA-C instability may exacerbate neurocognitive decline by promoting chronic immune activation and neuroinflammatory processes. Extensive scientific evidence supports the notion that genetic determinants of immune function are critical in shaping disease outcomes in HIV-1. In particular, previous studies have emphasized the role of HLA alleles in HIV-1 replication and progression (7, 10, 24, 25). Our findings extend this knowledge by highlighting the relevance of HLA-C allele stability at a more granular level. The association between HLA-C stability and neurocognitive disorders also aligns with research on β_2_m in neurodegeneration (29), providing a potential molecular framework for future investigations into HIV-associated neurocognitive impairment.

### HLA-C stability: towards a precision medicine approach

A major advantage of this refined stability analysis is the ability to calculate a personalized HLA-C stability score for each patient. By integrating computational stability assessments, such as EL-scores and AUC-derived stability coefficients, with patient-specific genetic data, it is possible to develop a predictive model that can help stratify patients based on their risk of rapid disease progression or neurocognitive complications. This approach aligns with the broader movement toward personalized medicine, where treatments and monitoring strategies are tailored to an individual’s genetic profile. From a clinical perspective, the ability to predict disease progression based on HLA-C stability could lead to more targeted interventions. For instance, patients identified as having unstable HLA-C variants could be prioritized for early intervention strategies, including more intensive monitoring, earlier initiation of antiretroviral therapy, or adjunctive therapies targeting immune modulation. Furthermore, understanding the link between HLA-C instability and HAND could open new therapeutic avenues, such as early interventions targeting neuroinflammatory pathways. The use of HLA-C genotyping could improve personalized treatment strategies, identifying individuals at higher risk of rapid disease progression or HAND.

The study of HLA-C peptide binding stability is critical for understanding immune regulation and its implications in infectious diseases, cancer, and autoimmunity. Advances in computational modelling and experimental methodologies provide valuable insights into the structural and functional aspects of HLA-C stability. Future research should focus on refining predictive models, defining clinically relevant stability thresholds and exploring therapeutic interventions aimed at enhancing HLA-C-mediated immune responses.

The main limitation of the study was the inability to perform more precise typing at the second digit for several biological samples due to insufficient DNA for testing. This reduced the accuracy of the data obtained, necessitating the use of a frequency-weighted average of stability scores in some cases.

## Conclusions

HLA-C plays a crucial and multifaceted role in HIV-1 infection, influencing immune recognition, disease progression, and neurocognitive outcomes. Despite its lower surface expression compared to HLA-A and HLA-B, HLA-C contributes significantly to both adaptive and innate immune responses. Advances in genomic and immunological research continue to reveal the complex interactions between HLA-C and HIV-1, providing valuable insights into potential therapeutic and vaccine strategies. By using an innovative cutting-edge bioinformatic pipeline, we demonstrated that reduced HLA-C stability is associated with faster HIV-1 disease progression and a higher prevalence of HIV-associated neurocognitive disorders. Overall, the findings from this study emphasize that HLA-C stability analysis should become an integral part of HIV-1 disease management and research. Future studies should focus on refining predictive models for personalized stability scoring, validating these findings in larger, independent cohorts, to early identify those at greater risk of progression or developing neurocognitive symptoms and intervene early with the most appropriate treatment approaches to improve patient outcomes.

## Data Availability

The original contributions presented in the study are included in the article/supplementary material. Further inquiries can be directed to the corresponding author.
